# Exosomal-mediated transfer of OIP5-AS1 enhanced cell chemoresistance to trastuzumab in breast cancer via up-regulating HMGB3 by sponging miR-381-3p

**DOI:** 10.1515/med-2021-0249

**Published:** 2021-03-30

**Authors:** Qiang Yu, Yinmou Li, Shijun Peng, Jing Li, Xianxiong Qin

**Affiliations:** Department of Breast Surgery, The Central Hospital of Enshi Tujia and Miao Autonomous Prefecture, No. 158 Wuyang Street, Enshi 445000, Hubei, China

**Keywords:** OIP5-AS1, miR-381-3p, HMGB3, breast cancer, exosomes, chemoresistance

## Abstract

**Background:**

Long noncoding RNA OPA-interacting protein 5 antisense transcript 1 (OIP5-AS1) was confirmed to involve in the malignancy of breast cancer. However, whether exosomal OIP5-AS1 is implicated in trastuzumab resistance remains unclear.

**Methods:**

The IC_50_ value of cells to trastuzumab, cell proliferation, migration, and apoptosis was analyzed by cell counting kit-8 assay, colony formation assay, transwell assay, or flow cytometry, respectively. The expression of OIP5-AS1 and microRNA (miR)-381-3p was detected using quantitative real-time polymerase chain reaction. Exosomes were isolated by ultracentrifugation and qualified by nanoparticle tracking analysis software. Western blot was used to detect the protein levels of tumor susceptibility gene 101 (TSG101), CD81, CD63, or high-mobility group protein B3 (HMGB3). The interaction between miR-381-3p and OIP5-AS1 or HMGB3 was confirmed by dual-luciferase reporter assay and pull-down assay. *In vivo* experiments were conducted using murine xenograft models.

**Results:**

OIP5-AS1 was elevated in trastuzumab-resistant breast cancer cells, and OIP5-AS1 knockdown rescued trastuzumab sensitivity. Extracellular OIP5-AS1 was packaged into exosomes, which were secreted by trastuzumab-resistant cells, and could be absorbed by trastuzumab-sensitive cells in breast cancer. Importantly, intercellular transfer of OIP5-AS1 via exosomes enhanced trastuzumab resistance *in vitro.* OIP5-AS1 was a sponge of miR-381-3p; besides, miR-381-3p targeted HMGB3. Murine xenograft analysis showed exosomal OIP5-AS1 induced trastuzumab resistance *in vivo.* Exosomal OIP5-AS1 was dysregulated in the serum of breast cancer patients and might be a promising diagnostic biomarker in trastuzumab resistance.

**Conclusion:**

Intercellular transfer of OIP5-AS1 by exosomes enhanced trastuzumab resistance in breast cancer via miR-381-3p/HMGB3 axis, indicating a potential therapeutic strategy to boost the effectiveness of trastuzumab in resistant breast cancer patients.

## 1 Introduction

Breast cancer is the most commonly diagnosed female malignancy on a global scale, which is a serious threat to the health of women worldwide [1]. A cancer cell has a variety of receptors on its surface, which can be bound by chemicals to result in changes within cancer cell. HER2 is a member of the epidermal growth factor receptor family that occurs in about one-third of all breast cancers [2]; importantly, HER2 amplification causes aggressive cancer phenotype and poor clinical outcome [3,4]. Trastuzumab, a humanized antibody of HER2, is thought to be a successful strategy to block HER2 receptors when there is overexpression, thereby blocking growth of cancer and prolonging the overall survival of HER2+ breast cancer patients in adjuvant and metastatic settings [5,6]. Nevertheless, the response rate to trastuzumab-containing therapies still needs to be improved because of the development of acquired trastuzumab resistance [7]. Therefore, a better understanding on the mechanism of trastuzumab resistance is essential for developing new approaches to overcome trastuzumab resistance in breast cancer.

Recently, it has been documented that cells can communicate with each other via microvesicles [8]. Exosomes are one of the extracellular vesicles, contain lipids, protein, noncoding, or coding RNAs, can be secreted by numerous cell types, like cancer cells, and can be absorbed by other cells to transfer and exchange the cargo [9,10]; thus, exosomes are considered as potential modes of intercellular communication [11]. Recently, emerging evidence has revealed that exosomes potentially impact the therapeutic response of the recipient cells via the transfer of proteins and lncRNAs [12]. Long noncoding RNA OPA-interacting protein 5 antisense transcript 1 (OIP5-AS1), mapped on chromosome 15q15.1, is a novel identified and promising tumor-associated lncRNA. It has been demonstrated that OIP5-AS1 plays various roles in multiple cancers and contributes to deterioration of malignant tumors [13]. In breast cancer, OIP5-AS1 was found significantly up-regulated and functioned as an oncogene via regulating cell malignant phenotypes [14], whereas the role of OIP5-AS1 in trastuzumab resistance in breast cancer still needs to be explored.

Thus, we attempted to elaborate the functions of OIP5-AS1 in trastuzumab resistance in breast cancer, explored whether the exosome-transmitted OIP5-AS1 conferred drug resistance to recipient cells, as well as the potential molecular mechanism underlying OIP5-AS1 effects on breast cancer.

## 2 Materials and methods

### 2.1 Patients and specimens

Blood samples were collected from 57 breast cancer patients diagnosed by histopathological examination at The Central Hospital of Enshi Tujia and Miao Autonomous Prefecture. All blood samples were centrifuged at 3,000 g for 10 min after collecting for 1 h, and the supernatant serum was collected using RNase-free tubes and stored at −80°C until used. All patients only received trastuzumab-based neo-adjuvant chemotherapy and were classified into trastuzumab-resistant (non-response, *N* = 30) and trastuzumab-sensitive (response, *N* = 27) depending on the sensitivity to trastuzumab. This research was authorized by the Ethics Committee of The Central Hospital of Enshi Tujia and Miao Autonomous Prefecture and was carried out according to the guidelines of Declaration of Helsinki. Written informed consents had been collected from all subjects.

### 2.2 Cell culture

Human breast cancer cell lines SKBR3 and BT474 were purchased from Shanghai Academy of life Science (Shanghai, China) and cultured in RPMI-1640 medium (Gibco, Carlsbad, CA, USA) harboring with 15% fetal bovine serum (FBS) and 0.1 IU/mL insulin at 37°C with 5% CO_2_. Trastuzumab-resistant breast cancer cells, named SKBR3-TR and BT474-TR, were established by continuously exposing parental cells to increasing concentration of trastuzumab (Sigma, St. Louis, MO, USA) for more than 6 months until cells displayed resistance to trastuzumab. Trastuzumab-resistant cells were maintained in the same media supplemented with 3 μg/mL trastuzumab.

### 2.3 Cell viability assay

Parental or resistant cells (5,000 cells/well) were seeded in 96-well plates overnight. Following transfection or trastuzumab treatment (0, 0.3125, 0.625, 1.25, 2.5, 5, or 10 μg/mL) for additional 48 h, cells in 96-well plates were incubated with 10 μL cell counting kit-8 solution (Sigma) for 4 h at 37°C. The optical density at 450 nm was determined by a microplate reader, and the half-maximal inhibitory concentration (IC_50_) value of trastuzumab was assessed on the basis of the relative survival curve.

### 2.4 Cell transfection

The miR-381-3p mimic (miR-381-3p), small interfering RNA (siRNA) targeting OIP5-AS1 (si-OIP5-AS1#1, si-OIP5-AS1#2, si-OIP5-AS1#3), pcDNA3.1 OIP5-AS1 overexpression vector (oe-OIP5-AS1), pcDNA3.1 HMGB3 overexpression vector (HMGB3), and their corresponding negative control (miR-NC, si-NC, Vector) were synthesized by Genepharma (Shanghai, China). The transfection of cells was performed using Lipofectamine™ 2000 transfection reagent (Invitrogen, Carlsbad, CA, USA).

### 2.5 RT-PCR and real-time quantitative PCR (qPCR)

Whole-RNA extracts from parental or resistant cells were prepared using TRIzol reagent (Invitrogen), and exosomal RNAs were isolated with the exoRNeasy Midi Kit (Qiagen, Valencia, CA, USA) according to the standard procedure. Complementary DNA (cDNA) was synthesized using SuperScript III^®^ (Qiagen), and quantitative PCR was performed using SYBR Premix Ex Taq (Qiagen) on the Bio-Rad CFX96 Sequence Detection system (Bio-Rad, Hercules, CA, USA). The expression levels were detected by 2^−∆∆Ct^ method with glyceraldehyde 3-phosphate dehydrogenase (GADPH) or U6 small nuclear B noncoding RNA (U6) serving as an internal reference. The primer sequences were listed as follows: OIP5-AS1: F, 5′-TGCGAAGATGGCGGAGTAAG-3′ and R, 5′-TAGTTCCTCTCCTCTGGCCG-3′; miR-381-3p: F, 5′-TAATCTGACTATACAAGGGCAAGCT-3′ and R, 5′-TATGGTTGTTCTGCTCTCTGTCTC-3′; GADPH: F 5′-GAGAAACCTGCCAAGTATGATGAC-3′ and R 5′-GGAGTTGCTGTTGAAGTCAC-3′, U6: F, 5′-CTCGCTTCGGCAGCACA-3′ and R, 5′-AACGCTTCACGAATTTGCGT-3′.

### 2.6 Colony formation assay

Transfected parental or resistant cells (5,000 per well) suspended in RPMI-1640 medium with 0.5 μg/mL trastuzumab were seeded in 6-well plates. After 21 days of cultures at 37°C with 5% CO_2_, cell colonies were fixed with methanol and stained with 0.1% crystal violet. Finally, the number of visible colonies (≥50 cells) was counted.

### 2.7 Cells migration assay

The migratory capacity of cells was performed by a 24-well transwell chamber (8 μm; Corning Costar, Cambridge, MA). Transfected cells suspended in serum-free RPMI-1640 medium filled the top chambers. Then, 500 μL RPMI-1640 medium mixed with 10% FBS was added into the lower chambers. After 24 h, cells on the lower face of the membranes were fixed and stained. Finally, migrated cells in five random fields were counted with a microscope.

### 2.8 Flow cytometry

Annexin V-fluorescein isothiocyanate (FITC)/propidium iodide (PI) apoptosis detection kit (BD Biosciences, San Jose, CA, USA) was used to detect cell apoptosis. In brief, after transfection with the designed vector for 48 h, cells were interacted with 5 μL FITC annexin V and 10 μL PI. Finally, the apoptotic rate was measured by a flow cytometer.

### 2.9 Exosome (exo) isolation

Exosomes were isolated from serum samples or cells using ultracentrifuge method. Cell culture fluid from exosome-depleted medium or serum was centrifuged at 3,000 *g* for 30 min at 4°C to remove cell fragments. Then, the resulting supernatant was further centrifuged at 100,000 *g* for 70 min at 4°C and filtered using 0.22 μm filtration. Subsequently, pelleted exosomes were washed with PBS and centrifuged at 100,000 *g* for 70 min again. Finally, purified exosomes were resuspended in PBS for the detection of the size and quality of exosomes using nanoparticle tracking analysis (NTA) software or for functional assays. Exosomes pellets were interacted with Trizol reagent to isolate RNA and were lysed with RIPA lysis buffer used for protein detection. For blocking of exosome release, parental or resistant breast cancer cells were treated with GW4869 (10 μM) or vehicle (as control) for 48 h. For exosome co-cultures, exosomes (50 μg/mL) were incubated with parental SKBR3 and BT474 cells (5 × 10^5^) in a 6-well plate with 10% FBS exosome-depleted culture medium for 48 h.

### 2.10 Transmission electron microscopy (TEM)

Purified exosomes were dropped on the carbon-coated copper grid and allowed to absorb for 5 min at 37°C and then stained with 2% phosphotungstic acid solution for 2 min, followed by washing with PBS thrice. After air-dried, the grid was visualized using a transmission electron microscope (TEM) (JEOL, Akishima, Japan).

### 2.11 Western blot

Proteins were extracted from cells or exosomes using RIPA lysis buffer (Beyotime, Beijing, China) and quantified and determined using a bicinchoninic acid Protein Assay Kit (Beyotime). Extractive protein was loaded on sodium dodecyl sulfate polyacrylamide gel electrophoresis for separation and then shifted onto polyvinylidene fluoride membranes. Later, membranes were interacted with CD81 (ab79559, 1:1,000, Abcam, Cambridge, MA, USA), CD63 (1:2,000, ab68418, Abcam), TSG101 (ab125011, 1:5,000, Abcam), HMGB3 (1:1,000, #6893, Cell Signaling Technology, Beverly, MA, USA), and the secondary HRP-conjugated antibody (1:1,000, ab9482, Abcam). The β-actin (1:1,000, #4970, Cell Signaling Technology) was used as an internal reference. The protein bands were visualized using the Image J software.

### 2.12 Dual-luciferase reporter assay

The predicted potential binding sequences of miR-381-3p in OIP5-AS1 and HMGB3 3′-untranslated (3′UTR) regions and their mutated sequence were separately cloned into pmirGLO Dual-luciferase vectors (Promega, Madison, WI, USA). Subsequently, these constructed vectors were co-transfected into SKBR3 and BT474 with miR-381-3p mimics or miR-NC using Lipofectamine™ 2000 (Invitrogen). The luciferase activities were detected using a dual luciferase assay kit (Promega).

### 2.13 Pull-down assay

Biotin (bio)-miR-381-3p and bio-NC synthesized by Genepharma Company were transfected into SKBR3 and BT474 for 48 h. Then, cells were lysed, and the lysates were incubated with M-280 streptavidin magnetic beads (Invitrogen). After elution, the bead-bound RNA complex was purified and subjected to qPCR analysis.

### 2.14 Xenograft experiments *in vivo*

Female BALB/c mice (5-week-old) from Jinan Pengyue Animal Center (Jinan, China) were randomly divided into four groups (*N* = 5 each). Each group was injected with BT474 cells (1 × 10^6^) in the flank region of mice. When tumors grew to 100 mm^3^, groups 2, 3, and 4 were intratumorally injected with trastuzumab (3 mg/kg) every 2 days, and the negative control of group 1 was injected with PBS; besides, isolated exosomes (10 μg) from BT474-TR cells loaded with si-OIP5-AS1 lentiviral vector (si-OIP5-AS1#1) or si-NC lentiviral vector (si-NC) were injected into the center of tumor of groups 4 and 3 every two days, respectively. Tumor volume was calculated every 4 days. At day 32, all mice were killed and tumor masses were weighed and harvested for further molecular analysis. Animal experimental protocols were permitted by the Animal Care and Use Committee of The Central Hospital of Enshi Tujia and Miao Autonomous Prefecture and performed in accordance with the guidelines of the National Animal Care and Ethics Institution.

### 2.15 Statistical analysis

Numerical results from three independent experiments were manifested as the mean ± standard deviation. The statistical difference between each group was analyzed by Student’s *t*-test or one-way analysis of variance with GraphPad Prism 7 software. Receiver operating characteristic (ROC) curves were plotted to analyze the diagnostic value of exosomal OIP5-AS1. *P*-values less than 0.05 were considered as statistically significant.

## 3 Results

### 3.1 OIP5-AS1 is up-regulated in trastuzumab-resistant breast cancer cells

**Figure 1:**
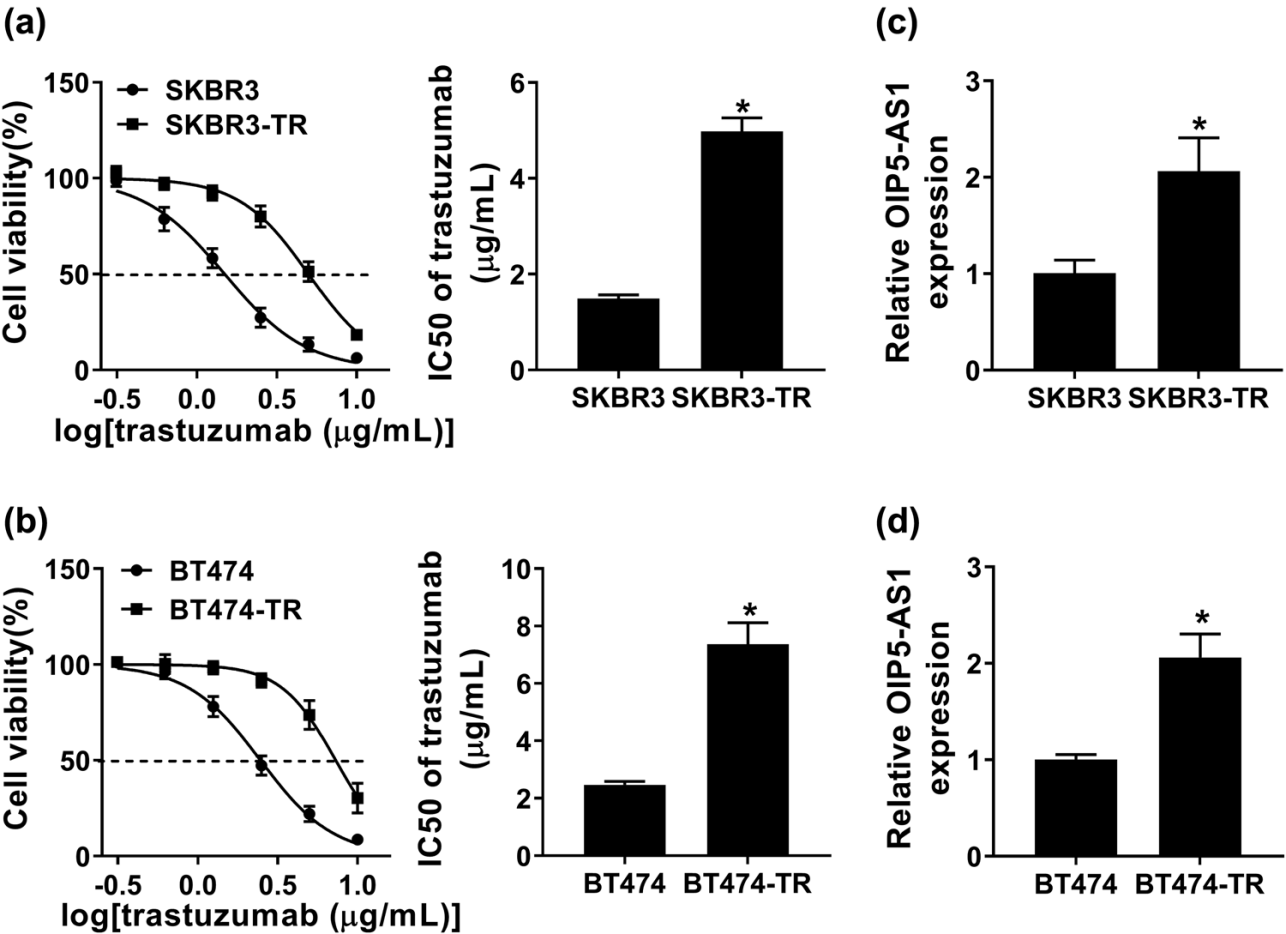
OIP5-AS1 is up-regulated in trastuzumab-resistant breast cancer cells. (a and b) CCK-8 Analysis of the viability of parental and resistant cell in combination with increasing concentrations of trastuzumab (0.3125–10 μg/mL) as well as the IC_50_ values of cells to trastuzumab. (c and d) Analysis of levels of OIP5-AS1 in SKBR3 and SKBR3-TR cells as well as in BT474 and BT474-TR cells. *P < 0.05.

To explore the role of OIP5-AS1 in trastuzumab resistance, trastuzumab-resistant breast cancer cells, named SKBR3-TR and BT474-TR, were established using the parental SKBR3 and BT474 cells to continuously expose with increasing concentration of trastuzumab for more than 6 months. Then, cell viability was detected in parental and resistant cells. Results showed the viability of parental SKBR3 and BT474 cells was significantly inhibited by trastuzumab in 0.3125–10 μg/mL compared with SKBR3-TR and BT474-TR cells, and the IC_50_ values of SKBR3-TR and BT474-TR cells to trastuzumab were markedly higher than that in SKBR3 and BT474 cells (Figure 1a and b); thus, SKBR3-TR and BT474-TR cells were defined as resistance. Later, the level of OIP5-AS1 was analyzed in breast cancer cells, and we found OIP5-AS1 was markedly higher in SKBR3-TR and BT474-TR cells than that in corresponding parental SKBR3 and BT474 cells (Figure 1c and d). These data indicated that the increase in OIP5-AS1 might be related to trastuzumab resistance in breast cancer.

### 3.2 OIP5-AS1 knockdown restores trastuzumab sensitivity in trastuzumab-resistant breast cancer cells

**Figure 2:**
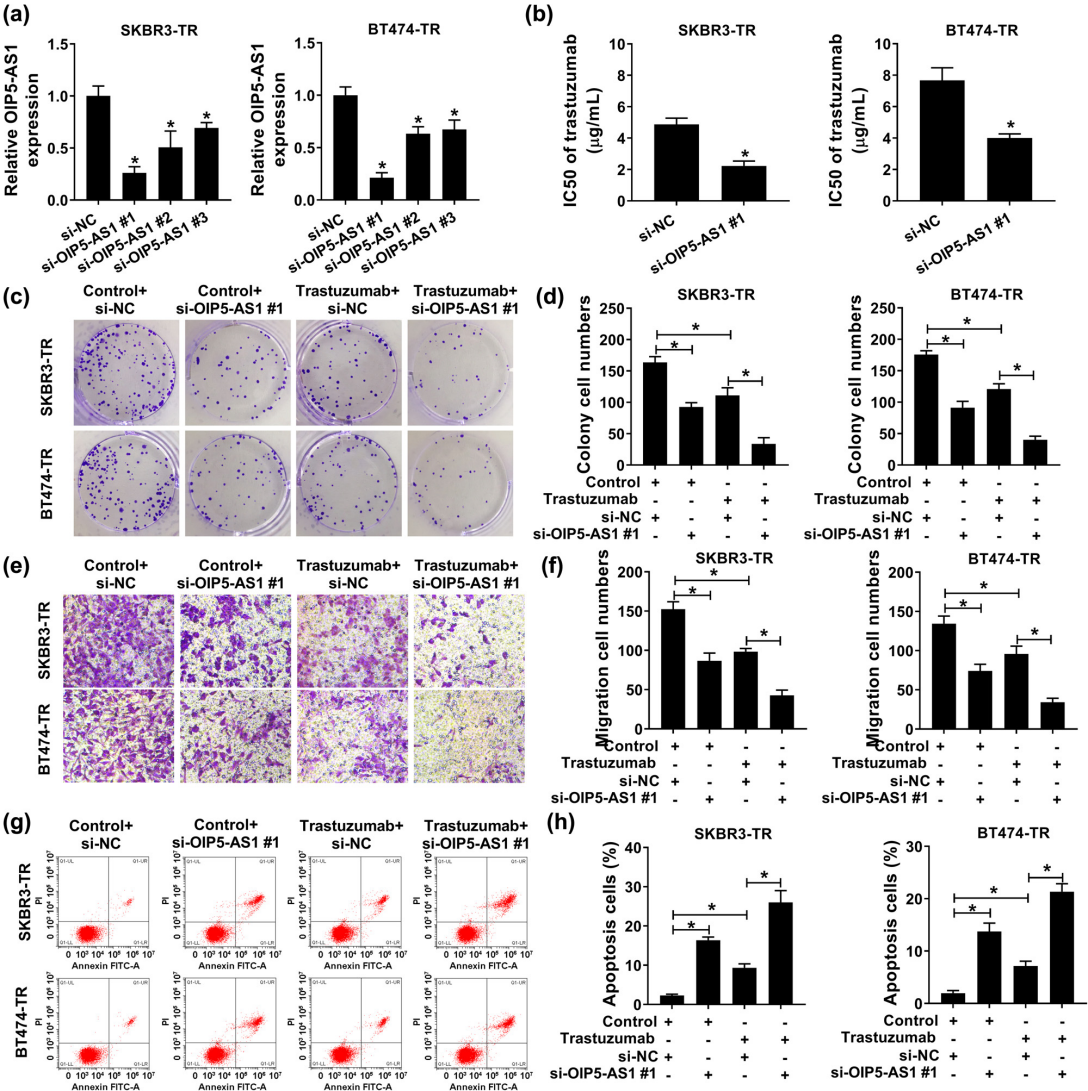
OIP5-AS1 knockdown restores trastuzumab sensitivity in trastuzumab resistant breast cancer cells. SKBR3-TR and BT474-TR cells were transfected with si-OIP5-AS1 or si-NC. After transfection, (a) qPCR analysis of OIP5-AS1 expression; (b) CCK-8 analysis of IC_50_ values of resistant cells to trastuzumab; (c and d) colony formation analysis of resistant cell proliferation; (e and f) transwell analysis of resistant cell migration; (g and h) resistant cell apoptosis analysis with flow cytometry. *P < 0.05.

To investigate the detailed functions of OIP5-AS1 in trastuzumab resistance, we knocked down OIP5-AS1 by transfecting with constructed si-OIP5-AS1 plasmid into SKBR3-TR and BT474-TR cells. As shown in Figure 2a, three forms of si-OIP5-AS1 all notably reduced OIP5-AS1 expression compared with the si-NC group, and then si-OIP5-AS1#1 was selected for subsequent analyses because of the efficient interference efficiency, which was reflected by the lowest OIP5-AS1 expression after si-OIP5-AS1 transfection in cells. By contrast with si-NC group, OIP5-AS1 down-regulation significantly reduced the IC_50_ values of SKBR3-TR and BT474-TR cells to trastuzumab (Figure 2b); in addition, colony formation analysis demonstrated that OIP5-AS1 knockdown combined with trastuzumab markedly decreased the number of colonies formed of SKBR3-TR and BT474-TR cells (Figure 2c and d). Meanwhile, transwell assay displayed the number of migrated and invaded SKBR3-TR and BT474-TR cells was significantly suppressed by the treatment of trastuzumab, and this reduction was markedly enhanced by the down-regulation of OIP5-AS1 (Figure 2e and f). Besides that, it was also proved that OIP5-AS1 depletion reinforced trastuzumab-induced elevation of the apoptosis of SKBR3-TR and BT474-TR cells (Figure 2g and h). Taken together, OIP5-AS1 knockdown impeded breast cancer cells to trastuzumab resistance.

### 3.3 Exosomal OIP5-AS1 derived from trastuzumab-resistant cells can be absorbed by trastuzumab-sensitive cells in breast cancer

**Figure 3:**
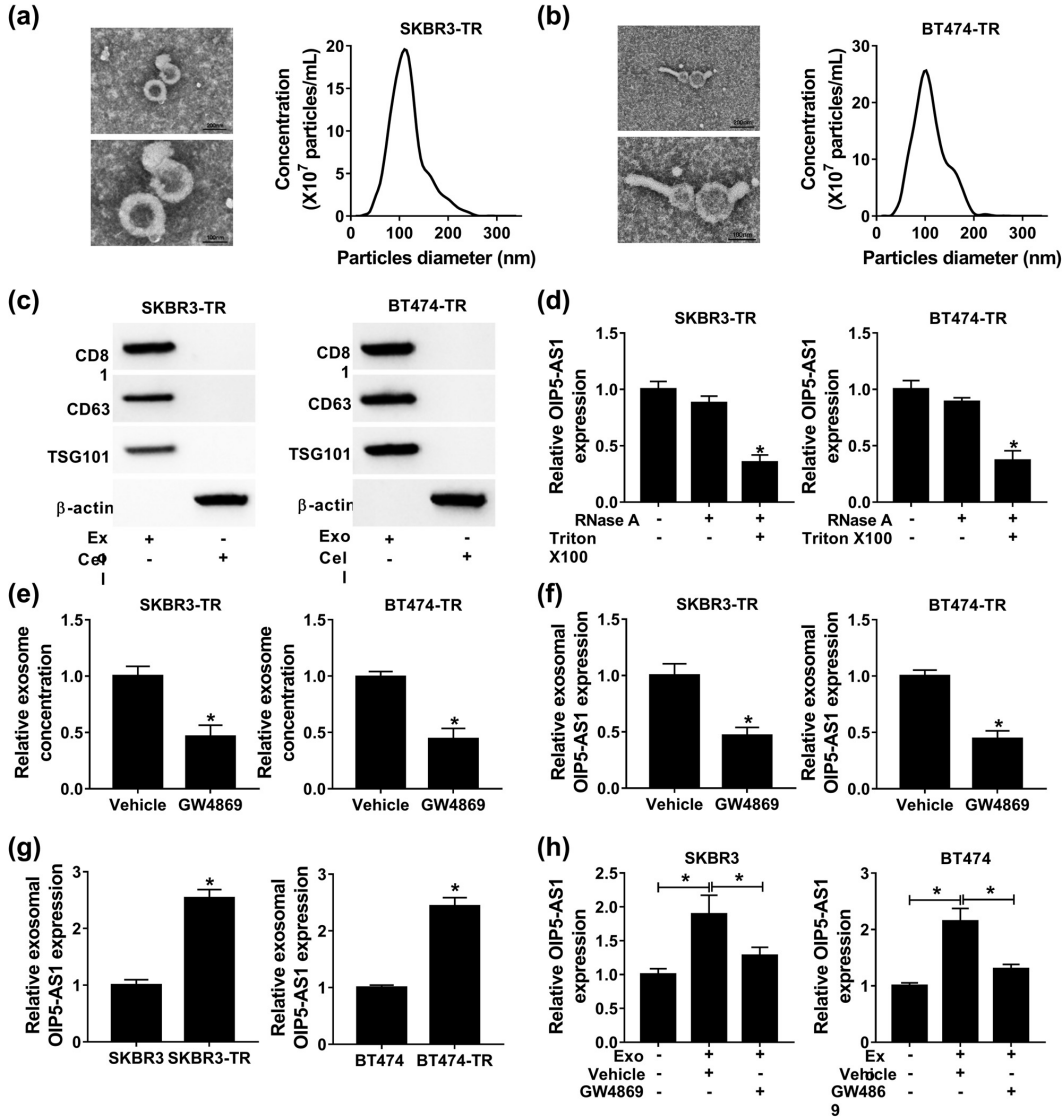
Exosomal OIP5-AS1 derived from trastuzumab resistant cells can be absorbed by trastuzumab-sensitive cells in breast cancer. (a and b) The image of purified exosomes derived from SKBR3-TR and BT474-TR cells captured by TEM; size distribution of purified exosomes was analyzed by NTA. (c) Western blot analysis of exosomal marker CD81, CD63, and TSG101 in isolated exosomes. (d) qPCR analysis of OIP5-AS1 expression in SKBR3-TR and BT474-TR cells following treatment with RNase alone or in combination with 0.1% Triton X-100. (e) The effect detection of GW4869 on the concentration of exosomes with a BCA protein assay. (f) qPCR analysis of OIP5-AS1 expression in exosomes treated with Vehicle and GW4869. (g) Analysis of levels of OIP5-AS1 in exosomes from SKBR3-TR and BT474-TR cells as well as parental SKBR3 and BT474 cells. (h) qPCR analysis of OIP5-AS1 expression in SKBR3 and BT474 cells co-cultured with exosomes, derived from SKBR3-TR and BT474-TR cells, and GW4869 or Vehicle. *P < 0.05.

Exosomes can be actively secreted by most cell types, and exosome-containing lncRNAs can be transmitted and exchanged into culture medium. To explore the impact of exosome transfer on trastuzumab resistance in breast cancer, we first isolated exosomes from the conditioned medium supernatant of SKBR3-TR and BT474-TR cells. The vesicles displayed a round shape with bilayered membranes, and the diameter from 40 to 250 nm under a TEM, and NTA analysis further confirmed that the predominant size of the vesicles was 100 nm (Figure 3a and b); in addition, as exhibited by western blot analysis, the exosomal markers CD63, CD81, and TSG101 were detectable in exosomes but not in cell lysates (Figure 3c). In addition, the expression change of OIP5-AS1 in the culture medium of SKBR3-TR and BT474-TR cells was detected after treatment with RNase. Results exhibited that OIP5-AS1 expression in culture medium was little affected upon RNase treatment but was greatly reduced when treated with RNase and Triton X-100 simultaneously (Figure 3d), indicating that OIP5-AS1 presented in exosomes. The treatment of SKBR3-TR and BT474-TR cells with GW4869, an inhibitor of the secretion of exosomes from cells, led to a reduction in the quantity of exosomes (Figure 3e) as well as OIP5-AS1 expression (Figure 3f). All these results indicated that extracellular OIP5-AS1 was packaged into exosomes, which were also secreted by SKBR3-TR and BT474-TR cells.

Next, exosomes from the culture supernatant of SKBR3 and BT474 cells were isolated, and qPCR analysis revealed OIP5-AS1 expression in SKBR3-TR and BT474-TR secreted exosomes was significantly higher than that in exosomes from parental cells (SKBR3 and BT474) (Figure 3g). Importantly, SKBR3 and BT474 cells co-cultured with exosomes derived from SKBR3-TR and BT474-TR cells were treated with GW4869 or vehicle, and we found the levels of OIP5-AS1 were increased by exosomes incubation but were decreased by GW4869 treatment (Figure 3h), revealing OIP5-AS1 could be transmitted into trastuzumab-sensitive cells via exosome transfer.

### 3.4 Intercellular transfer of OIP5-AS1 by exosomes enhances trastuzumab resistance *in vitro*

**Figure 4:**
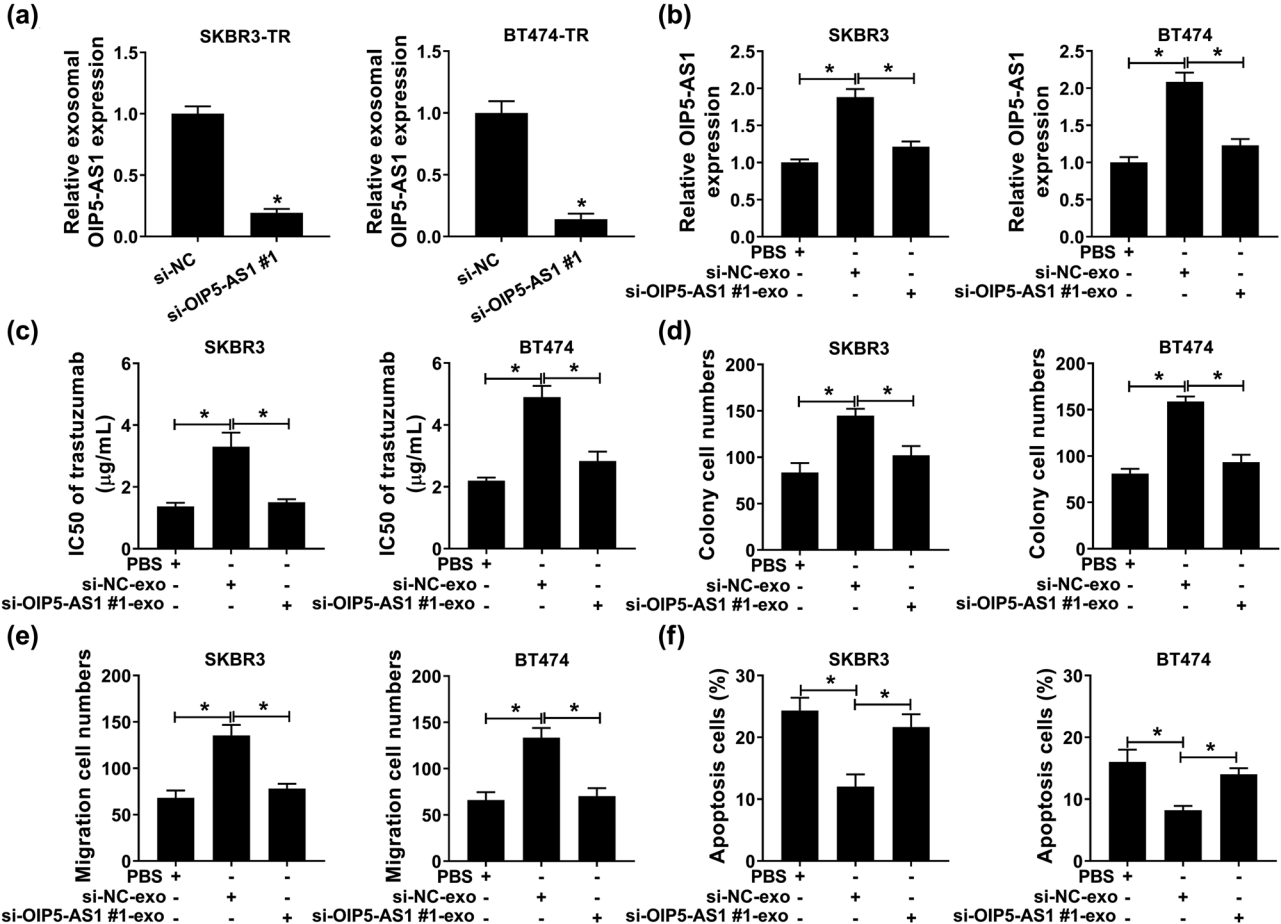
Intercellular transfer of OIP5-AS1 by exosomes enhances trastuzumab resistance *in vitro.* (a) qPCR analysis of OIP5-AS1 expression in exosomes isolated from SKBR3-TR and BT474-TR cells transfected with si-OIP5-AS1#1 or si-NC. SKBR3 and BT474 cells were co-cultured with exosome-depletion PBS, si-OIP5-AS1#1-exo, or si-NC-exo. After incubation; (b) levels of OIP5-AS1 were detected with qPCR; (c) CCK-8 analysis of IC_50_ values of parental cells to trastuzumab; (d) colony formation analysis of parental cell proliferation; (e) migration analysis of parental cells using transwell assay; (f) flow cytometry assay for parental cell apoptosis. *P < 0.05.

To identify whether OIP5-AS1 conferred trastuzumab resistance through the delivery of exosomes, first, exosomes were isolated from SKBR3-TR and BT474-TR cells transfected with si-OIP5-AS1#1 or si-NC, and qPCR revealed a high expression of OIP5-AS1 in exosomes from donor SKBR3-TR and BT474-TR cells transfected with si-NC (si-NC-exo) compared with si-OIP5-AS1#1 (si-OIP5-AS1#1-exo) (Figure 4a). Second, SKBR3 and BT474 cells were co-cultured with exosome-depletion PBS, si-OIP5-AS1#1-exo or si-NC-exo, and we found OIP5-AS1 expression was elevated with si-NC-exo incubation relative to PBS treatment, while si-OIP5-AS1#1-exo incubation reduced the level of OIP5-AS1 in recipient cells (Figure 4b). Subsequently, the two recipient cell lines exhibited increased IC_50_ values (Figure 4c), elevated cell viability (Figure 4d), migration (Figure 4e), and decreased apoptosis (Figure 4f) following treatment with si-NC-exo compared with PBS group. By contrast with the si-NC-exo group, proliferation and migration of cells declined and apoptosis raised in the si-OIP5-AS1#1-exo group (Figure 4c–f). Altogether, these data revealed that SKBR3 and BT474 cells with exosomal OIP5-AS1 exhibited resistance to trastuzumab resistance.

### 3.5 OIP5-AS1 is a sponge of miR-381-3p

**Figure 5:**
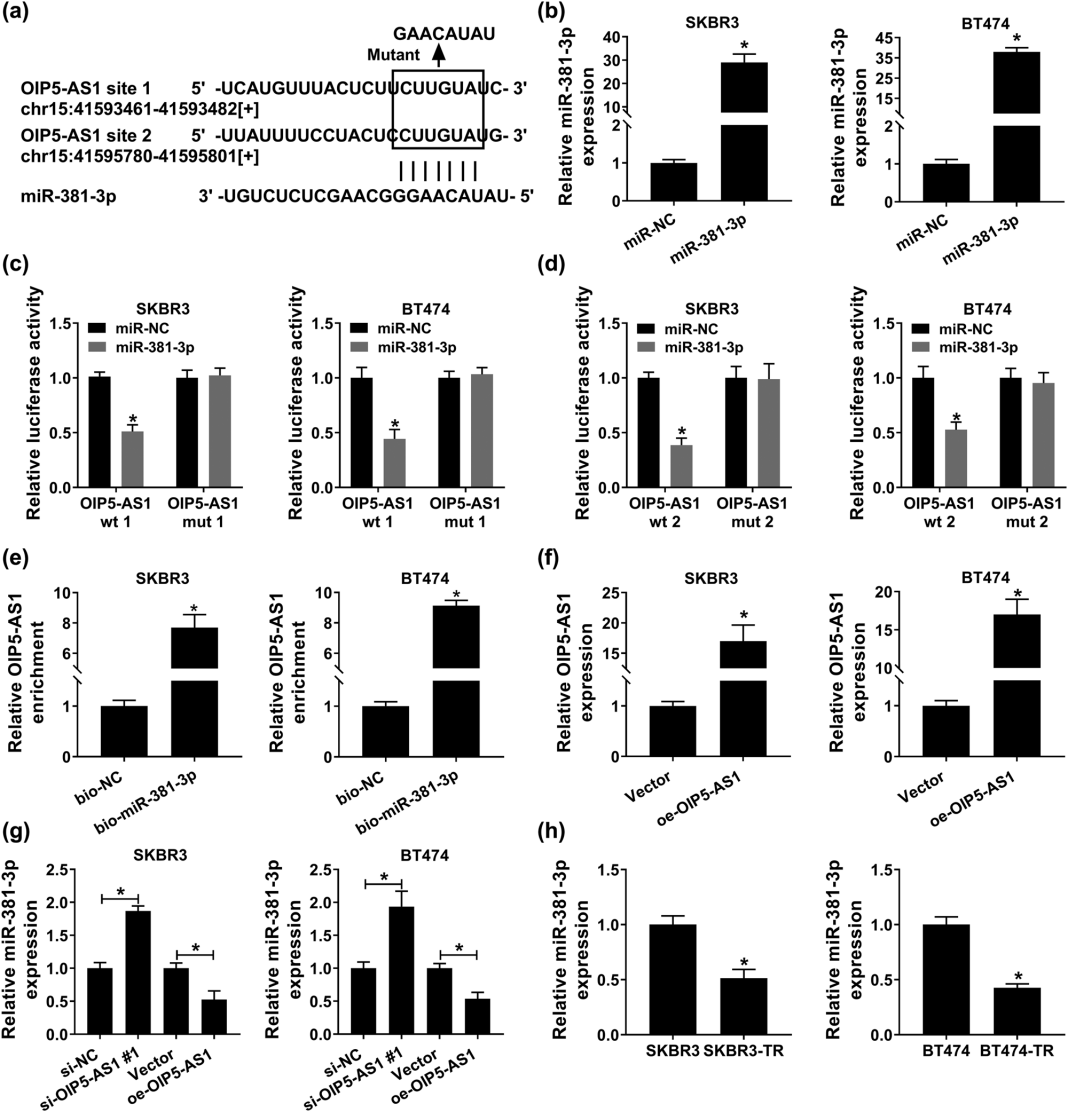
OIP5-AS1 is a sponge of miR-381-3p. (a) The potential binding sites of OIP5-AS1 and miR-381-3p. (b) qPCR analysis of miR-381-3p in SKBR3 and BT474 cells transfected with miR-NC or miR-381-3p. (c and d) Dual-luciferase reporter assay in SKBR3 and BT474 cells co-transfected with the reporter plasmid and the indicated miRNAs. (e) qPCR analysis of the enrichment level of OIP5-AS1 pulled down by bio-miR-381-3p or bio-NC in SKBR3 and BT474 cells. (f) qPCR analysis of OIP5-AS1 in SKBR3 and BT474 cells transfected with oe-OIP5-AS1 or Vector. (g) qPCR analysis of miR-381-3p expression in SKBR3 and BT474 cells transfected with oe-OIP5-AS1, Vector, si-NC, or si-OIP5-AS1#1. (h) Detection of levels of miR-381-3p in parental and resistant breast cancer cells with qPCR. *P < 0.05.

To explore the underlying mechanism of OIP5-AS1, miRNA targets were searched through starBase program, and miR-381-3p was found that might be a target of OIP5-AS1 (Figure 5a). To confirm this prediction, first, SKBR3 and BT474 cells were transfected with miR-NC or miR-381-3p, and miR-381-3p expression was significantly elevated by miR-381-3p transfection compared with miR-NC (Figure 5b). Then, a dual luciferase reporter assay was conducted and results showed that the luciferase activity of OIP5-AS1 wt of both forms was decreased in SKBR3 and BT474 cells treated with miR-381-3p; however, there was no significant difference in luciferase activity on the two forms of OIP5-AS1 mut (Figure 5c and d). Besides that, the results of pull-down assay revealed that the enrichment level of OIP5-AS1 in the bio-miR-381-3p group was markedly higher than that in the bio-NC group (Figure 5e). All these results suggested OIP5-AS1 specifically bound miR-381-3p. Next, SKBR3 and BT474 cells were transfected with oe-OIP5-AS1 to elevate OIP5-AS1 (Figure 5f), and qPCR analysis exhibited that miR-381-3p expression was decreased by OIP5-AS1 overexpression, but was increased by OIP5-AS1 down-regulation in SKBR3 and BT474 cells (Figure 5g). Thus, OIP5-AS1 targetedly repressed miR-381-3p expression in breast cancer cells. In addition, miR-381-3p was found to be decreased in SKBR3-TR and BT474-TR cells relative to parental SKBR3 and BT474 cells (Figure 5h), indicating miR-381-3p was associated with trastuzumab resistance in breast cancer.

### 3.6 HMGB is a target of miR-381-3p

**Figure 6:**
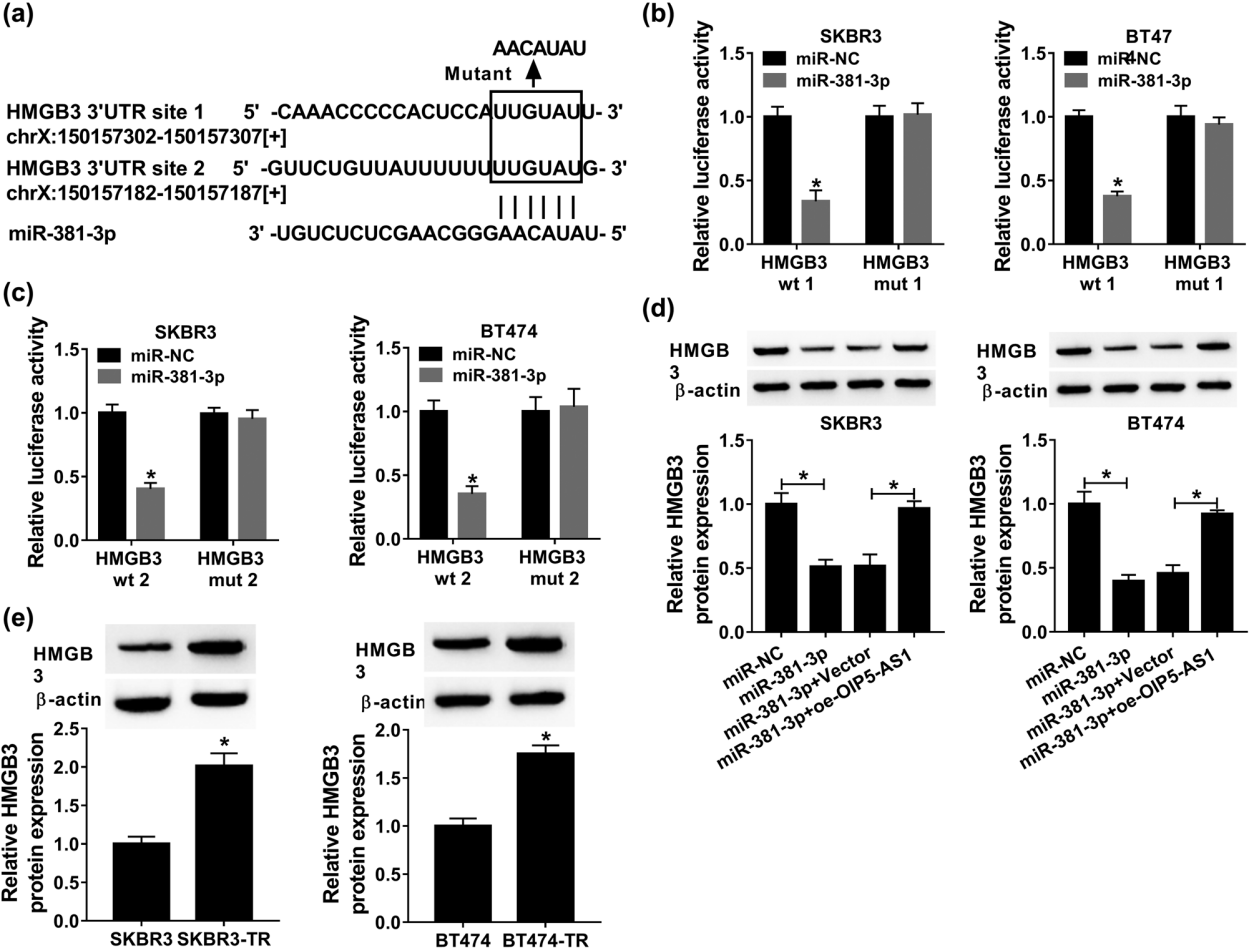
HMGB is a target of miR-381-3p. (a) Schematic representation of the predicted binding sites of miR-381-3p on HMGB. (b and c) Dual-luciferase reporter assay in SKBR3 and BT474 cells co-transfected with the reporter plasmid and the indicated miRNAs. (d) Western blot analysis of HMGB expression in SKBR3 and BT474 cells transfected with miR-NC, miR-381-3p, miR-381-3p + Vector, or miR-381-3p + OIP5-AS1. (e) Western blot analysis of HMGB expression in parental and resistant breast cancer cells. *P < 0.05.

According to the prediction of starBase program, miR-381-3p was found to have the binding sites on HMGB (Figure 6a). The results of dual luciferase reporter assay displayed that miR-381-3p overexpression reduced the luciferase activity of HMGB wt of both forms but not mutant reporter vector in SKBR3 and BT474 cells (Figure 6b and c), indicating that miR-381-3p directly targeted to HMGB. Meanwhile, western blot analysis exhibited that miR-381-3p restoration suppressed HMGB expression, while this inhibition was reversed by OIP5-AS1 overexpression in SKBR3 and BT474 cells (Figure 6d). Therefore, miR-381-3p targetedly suppressed HMGB expression and OIP5-AS1 positively regulated HMGB via miR-381-3p. Interestingly, HMGB was increased in SKBR3-TR and BT474-TR cells compared with their parental SKBR3 and BT474 cells (Figure 6e); thus, HMGB was also linked to trastuzumab resistance in breast cancer.

### 3.7 Exosomal OIP5-AS1 induces trastuzumab resistance and promotes tumor growth *in vivo*

**Figure 7:**
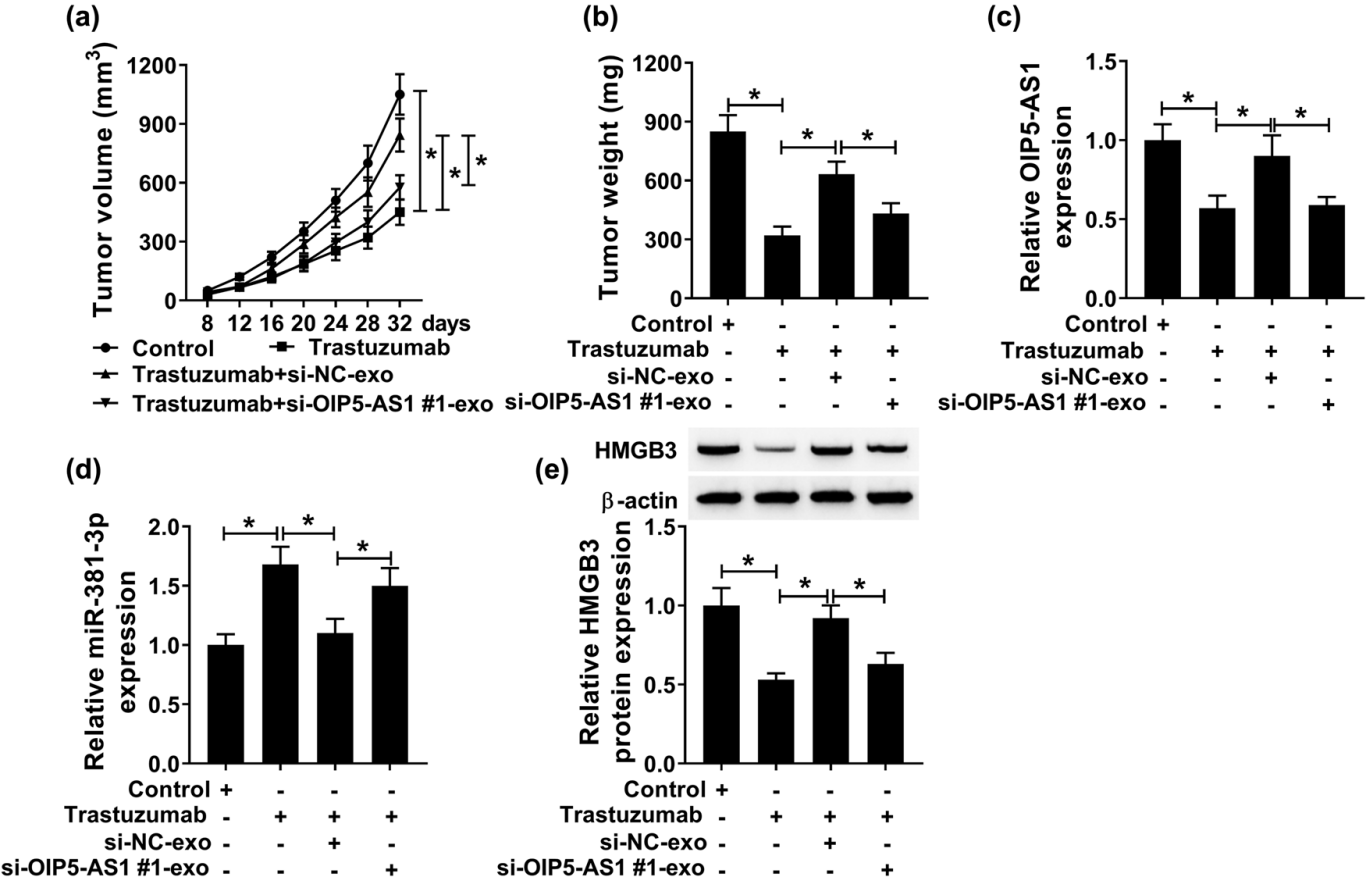
Exosomal OIP5-AS1 induces trastuzumab resistance and promotes tumor growth *in vivo.* (a) Tumor volumes were calculated every 4 days. (b) Tumor masses were collected and weighed on day 32. (c and d) Detection of levels of OIP5-AS1 and miR-381-3p in the tumor messes of each group with qPCR. (e) Western blot analysis of HMGB protein in the tumor messes of each group. *P < 0.05.

The effect of OIP5-AS1 on trastuzumab resistance *in vivo* was determined. As shown in Figure 7a and b, trastuzumab treatment significantly inhibited tumor growth in nude mice compared with control groups (group 1 vs group 2). More importantly, with trastuzumab treatment, tumor cells in si-NC-exo group grew faster than trastuzumab or si-OIP5-AS1#1-exo group (group 3 vs group 2 or group 4, respectively) (Figure 7a and b), suggesting that exosome-mediated transfer of OIP5-AS1 suppressed the cytotoxicity induced by trastuzumab treatment *in vivo*. In addition, molecule analysis showed si-NC-exo had high levels of OIP5-AS1 compared with si-OIP5-AS1#1-exo, and attenuated trastuzumab treatment-induced OIP5-AS1 reduction in tumors (Figure 7c). Besides, si-NC-exo with high OIP5-AS1 expression decreased miR-381-3p and elevated HMGB in tumors, while these effects were antipodal in si-OIP5-AS1#1-exo (Figure 7d and e). Collectively, exosomal OIP5-AS1 might induce trastuzumab resistance and promote tumor growth via regulating miR-381-3p and HMGB *in vivo*.

### 3.8 Serum exosomal OIP5-AS1 level is associated with trastuzumab resistant in breast cancer patients

**Figure 8:**
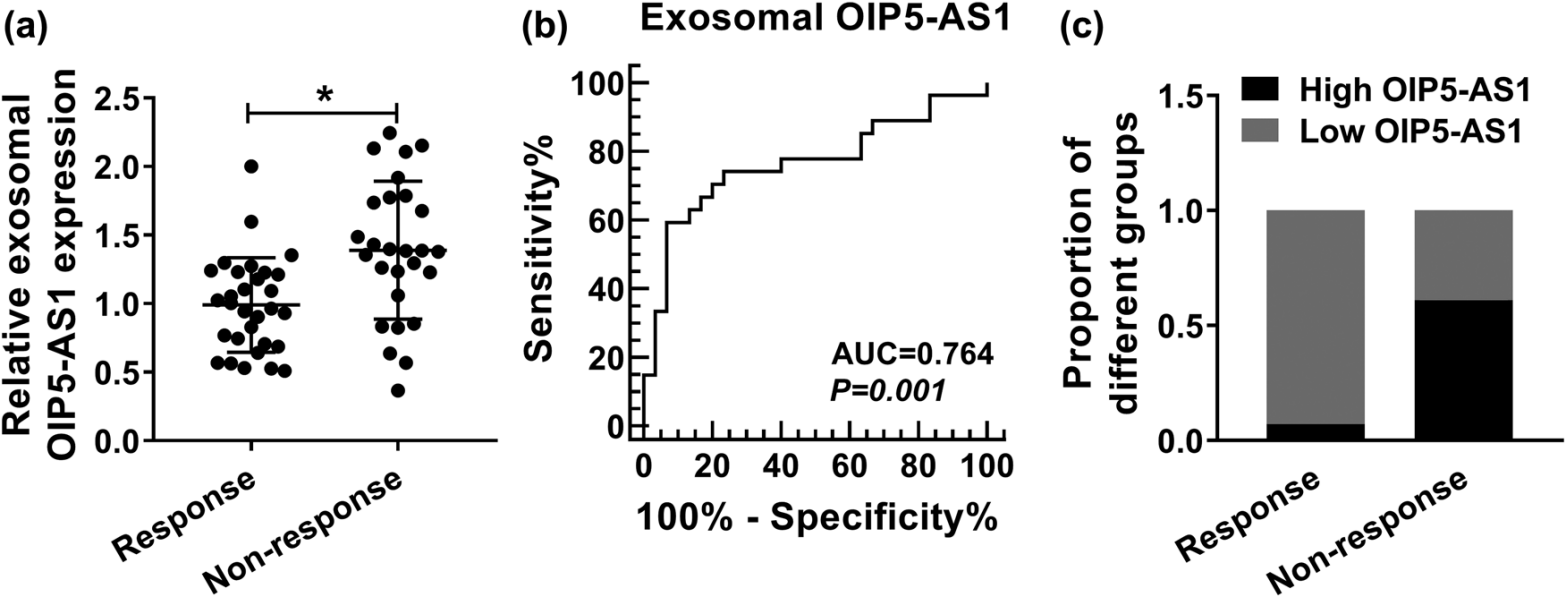
Serum exosomal OIP5-AS1 level is associated with trastuzumab resistant in breast cancer patients. (a) qPCR analysis of OIP5-AS1 in exosomes isolated from the serum of breast cancer patients responding or not responding to trastuzumab treatment. (b) ROC curve analysis of the diagnostic value of exosomal OIP5-AS1 in breast cancer patients receiving trastuzumab treatment. (c) qPCR suggested that the proportion of patients not responding to trastuzumab was greatly higher in the high exosomal OIP5-AS1 expressing group than in the low expressing group. *P < 0.05.

We further attempted to analyze the expression level of exosomal OIP5-AS1 in 57 serum samples from breast cancer patients receiving trastuzumab treatment. Results implied that the serum exosomal OIP5-AS1 remarkably higher in patients who did not respond to treatment than in those who responded to trastuzumab (Figure 8a). Later, the diagnostic potential of exosomal OIP5-AS1 in serum was calculated. As revealed by ROC analysis, an area under curve of 0.764 with a diagnostic sensitivity and specificity reaching 59.26 and 93.33%, respectively (95% CI = 0.6326–0.8958), was observed (Figure 8b). In addition, patients were classified into a low and high exosomal OIP5-AS1 expression groups according to the cut-offs (1.355) established by ROC, and we found the proportion of patients not responding to trastuzumab was greatly higher in the high exosomal OIP5-AS1 expressing group than in the low expressing group (Figure 8c). These data indicated that exosomal OIP5-AS1 was also dysregulated in the serum of breast cancer patients and might be a promising diagnostic biomarker for trastuzumab resistance in patients with breast cancer.

## 4 Discussion

Molecular targeted therapies are one of the major and revolutionized modalities of medical treatment, which interfere with specific molecules to block cancer growth, survival, and progression [15]. In breast cancer, some molecular targeted therapies, including trastuzumab and lapatinib, have been approved and demonstrated remarkable clinical success in the treatment of cancer. They direct against HER2 and bevacizumab as well as vascular endothelial growth factor [16,17]. lncRNAs are a class of RNAs with lengths exceeding 200 nucleotides and have been widely revealed to implicate in various physical and pathological processes, such as cell differentiation, proliferation, apoptosis, and metabolism, thereby affecting the development and progression of cancers [18,19,20,21]. They are considered as candidate therapeutic targets.

OIP5-AS1 is a functional RNA and has been identified to sever as an oncogene to participate in the evolution of tumorigenesis and drug resistance in many types of cancer. For example, OIP5-AS1 accelerated the progression of gastric cancer by regulating HMGA2 through miR-367-3p [22]. OIP5-AS1 interacted with ROCK1 to promote cell carcinogenesis in cervical cancer via absorbing miR-143-3p. OIP5-AS1 induced cisplatin resistance in osteosarcoma through regulating the LPAATβ/PI3K/AKT/mTOR signaling pathway via a mechanism involving miR-340-5p [23]. Importantly, OIP5-AS1 was confirmed to act as an oncogene via regulating cell malignant phenotypes in breast cancer [14]. Thus, we thought OIP5-AS1 might also involve in the regulation of drug resistance and then confirmed that knockdown of OIP5-AS1 restored trastuzumab sensitivity in trastuzumab-resistant breast cancer cells.

Exosomes are one of the extracellular vesicles and have been reported to involve in the modulation of chemoresistance of the recipient cells via the transfer of lncRNAs in diverse cancers [24,25]. In breast cancer, the effects of exosomal lncRNA in drug resistance were also demonstrated. For instance, exosome-mediated transfer of AFAP1-AS1 induced trastuzumab resistance in breast cancer via the interaction with AUF1 and the activation of ERBB2 translation [26]. Intercellular transfer of lncRNA H19 promoted doxorubicin resistance in breast cancer [27]. Thus, whether exosomal OIP5-AS1 is implicated in trastuzumab resistance in breast cancer was investigated. Results exhibited that extracellular OIP5-AS1 was packaged into exosomes, which were secreted by trastuzumab-resistant cells and could be taken up by trastuzumab sensitive cells via exosomes transfer, thereby disseminated trastuzumab resistance to recipient cells *in vitro* as well as inhibited cytotoxicity induced by trastuzumab in tumor growth *in vivo* in breast cancer. In addition, exosomal OIP5-AS1 was also dysregulated in the serum of breast cancer patients and might act as a promising diagnostic biomarker for trastuzumab resistance in breast cancer patients. Therefore, exosomal OIP5-AS1 may be useful for the treatment of breast cancer and the prediction of trastuzumab resistance. The potential underlying molecular mechanism of the action of OIP5-AS1 was further probed. In this study, we found OIP5-AS1 directly bound to miR-381-3p and subsequently served as a miRNA sponge to up-regulate the expression of the miR-381-3p target gene HMGB3. miR-381-3p is a well-recognized anti-tumor miRNA, the overexpression of which was confirmed to suppress cell proliferation, cell cycle progression, and migration in breast cancer [28]. HMGB3 belongs to HMGB family and plays significant effects on DNA repair, recombination, transcription, and replication [29]. Besides that, it has been demonstrated that HMGB3 silence could inhibit cell growth and progression in breast cancer [30,31]. Thus, we considered that exosomal OIP5-AS1 may confer trastuzumab resistance in breast cancer cells via the regulation of miR-381-3p/HMGB3 axis.

In conclusion, this study demonstrated that exosome-mediated transfer of OIP5-AS1 partially induced trastuzumab resistance in breast cancer through miR-381-3p/HMGB3 axis, indicating a therapeutic strategy for the trastuzumab resistance in patients with breast cancer.
